# Association between body mass index and diabetes mellitus control classification among patients with type 2 diabetes mellitus: Evidence from the National Diabetes Registry of Muar District Health Office, Malaysia, from January 2021 to July 2023

**DOI:** 10.51866/oa.794

**Published:** 2025-04-12

**Authors:** Muhammad Muzzammil Mohamad Salleh, Mohamad Rodi Isa, Mazapuspavina Md. Yasin, Nazar Mohd Azahar, Mohd Ridzuan Mohd Lutpi

**Affiliations:** 1 MBBS, DAP&E, MPH, DrPH, Department of Public Health, Faculty of Medicine, Universiti Teknologi MARA, Sungai Buloh, Selangor, Malaysia. Email: rodi@uitm.edu.my; 2 MBBCh, MPH, Department of Public Health, Faculty of Medicine, Universiti Teknologi MARA, Sungai Buloh, Selangor, Malaysia.; 3 MBBS, MMed (Fam Med), Department of Family Medicine, Faculty of Medicine, Universiti Teknologi MARA, Sungai Buloh, Selangor, Malaysia.; 4 BSc (Biomedical Science), MSc (Medicine), PhD, Department of Medical Laboratory Technology, Faculty of Health Sciences, Universiti Teknologi MARA (UiTM) Cawangan Pulau Pinang, Kampus Bertam, Pulau Pinang, Malaysia.; 5 MBBS, DrPH, Segamat District Health Office, Jalan Gudang Ubat, Kampung Gubah, Segamat, Johor Darul Ta'zim, Malaysia.

**Keywords:** Body mass index, Diabetes mellitus control, Type 2 diabetes mellitus

## Abstract

**Introduction:**

Type 2 diabetes mellitus (T2DM) is one of the most prevalent non-communicable diseases globally. This study aimed to determine the association between body mass index (BMI) and diabetes mellitus (DM) control among patients with T2DM.

**Methods:**

A retrospective study was conducted from October 2023 to June 2024 using secondary data from the National Diabetes Registry (NDR) of Muar District Health Office, Johor. Patients with T2DM registered in the NDR and audited from 2021 to July 2023 were included. The association between BMI and DM control was analysed using hierarchical multinomial logistic regression.

**Results:**

A total of 1955 patients were included in the study. The prevalence of good, intermediate and poor BMI control was 38.8% (95% Confident Interval (CI)=36.7, 41.0), 22.2% (95% CI=20.3, 24.0) and 39.0% (95% CI=36.7, 41.2), respectively. Most patients were older Malay women. There was an association between BMI and DM control unadjusted (P<0.001) and adjusted for several confounding factors using seven models (P=0.003-0.034). The R^2^ value also improved from 0.008 to 0.293. Conclusion: Among patients with T2DM, a higher BMI, the creatinine level and medications such as glucose-lowering drugs, ticlopidine, acetylsalicylic acid and statins are associated with DM control. However, as the study design does not allow for the assessment of causality or progression over time, the findings should be interpreted as descriptive associations rather than as evidence of cause-and-effect relationships. Focus on medication compliance, healthcare providers’ role during medication consultation and stakeholders’ role in maintaining drug supplementation is needed.

## Introduction

Diabetes mellitus (DM) is characterised by persistently high blood sugar levels and is directly linked to several major health problems including neuropathy, nephropathy, retinopathy and obesity. Within Asian populations, the prevalence of type 2 diabetes mellitus (T2DM) has increased from 8.1% to 9.6%, while within Caucasian populations, it has increased from 6.0% to 7.9%.^1^ The prevalence of T2DM has also become a worldwide public health concern that poses a threat to the economies of all countries, particularly developing nations.^2^ Increasingly sedentary lifestyle, growing urbanisation and a transition in nutrition have all contributed to the growth of the epidemic, which has occurred concurrently with the rise in obesity rates worldwide. The Malaysian National Health and Morbidity Survey (NHMS) in 2019 indicated that the prevalence of T2DM in Malaysia climbed to 18.3% from 17.5% in 2015.^3^ In the 2019 NHMS, the prevalence of DM in Johor, Malaysia, was 19.7%, which was higher than the 18.3% overall prevalence in the country. Additionally, the prevalence of obesity among adults aged 18 years in Johor was 35.6%, higher than the 33.7% overall prevalence in Malaysia.^3^

DM is a persistent metabolic disorder that manifests itself through elevated levels of glucose in the bloodstream, accompanied by impairments in the function of pancreatic β-cells and insulin resistance, which are the two primary processes that contribute to the development of this condition.^4^ According to Evans et al.,^5^ the glycated haemoglobin A1c (HbA1c) level is an essential measurement for determining overall glycaemic control in patients with diabetes. This measurement has been suggested by the American Diabetes Association for evaluating long-term glycaemic management and is commonly used as a standard measurement for this purpose.^6^ According to Xing et al.,^6^ the HbA1c level is a helpful indicator of the status of glycaemic control. Levels lower than 7.0% are generally considered to be indicative of good glycaemic control. Despite this, extreme glycaemic control that is exclusively based on the HbA1c level has the potential to result in severe hypoglycaemia and death from cardiovascular disease.

DM control is classified into three categories: good (HbA1c level of <7.0%), intermediate (HbA1c level of 7.0%–7.9%) and poor (HbA1c level of ≥8%).^7^ The HbA1c level reflects the status of DM control and has shown a good correlation with anthropometric measurements.^8^ Most patients (nearly 84%) with T2DM in Malaysia have a body mass index (BMI) of >23 kg/m^2^, which may also contribute to DM control. According to Fekadu et al.,^9^ several factors contributing to DM control have been identified, such as medication used, diet intake, physical activities and disease duration. However, these variables are difficult to assess and invasively measured, and some of them could be either true factors or just confounding factors contributing to glycaemic control. Therefore, this study aimed to determine the association between DM control (HbA1c level) and BMI adjusted for other important variables among patients with T2DM. BMI is a parameter commonly used in patient follow-ups. It can be non-invasively and easily collected. Hence, this study is expected to enhance knowledge regarding the association between BMI and glycaemic control, particularly in the local context. Furthermore, the Clinical Practice Guideline (CPG) for Managing Diabetes Mellitus provides specific HbA1c targets for different populations rather than a single standard for all.

## Methods

This retrospective study was conducted from October 2023 to June 2024 using secondary data from the National Diabetes Registry (NDR) of Muar District Health Office (PKD Muar).

The NDR was established in 2009 to monitor the clinical outcomes of patients with diabetes managed at the primary health clinics of the Ministry of Health, Malaysia.^10^ All patients with diabetes who receive diabetes care at participating health clinics are required to be registered in this registry. A proportion of patient records are audited annually, and all information about clinical care and treatment is stored in the NDR.^10^

The study population was patients with T2DM registered in the NDR under PKD Muar and audited from 2021 to July 2023. We excluded patients with missing values for the HbA1c level and BMI. For patients who had multiple follow-up visits, we included patient information from the last visit only.

### Data management

The study variables were sociodemographic characteristics (age, sex, ethnicity and smoking status), anthropometric measurements (height, weight, systolic blood pressure and diastolic blood pressure), medical illnesses (hypertension status and dyslipidaemia status), biochemical profile (HbA1c level, total cholesterol [TC] level, triglyceride [TG] level, high-density lipoprotein [HDL] level, low-density lipoprotein [LDL] level, serum creatinine level and proteinuria) and medications (metformin, sulfonylurea, insulin, acetylsalicylate acid, ticlopidine, ACE inhibitor, angiotensin-receptor blocker, beta-blocker, calcium-channel blocker, diuretic, alpha-blocker, statin, fibrate and alpha-glucosidase inhibitor).

There were 13 variables without missing values and 11 variables with missing values (0.1%–95.9%). Missing values were imputed by replacing numerical data with the mean of the variable and categorical data with linear extrapolation; values more than 0.5 were assigned a value of 1, and those less than 0.5 were assigned a value of 0.

The removed variables were other antiplatelet drugs besides ticlopidine and acetylsalicylic acid (high missing values: 95.9%), albuminuria status (high missing values: 18.9%), waist circumference (almost similar variable with BMI that may lead to multicollinear issue), glitazone (0 values in the cell) and meglitinides (no medications used for the variables).

Several variables were created based on the available data, including the duration of DM (measured by subtracting age from age during DM diagnosis) and BMI (measured by dividing weight in kilogrammes by height in metres square). DM control was classified into good, intermediate and poor.

BMI was treated as continuous data rather than as categorical data to better observe trends, as categorising BMI can obscure such patterns. A cutoff point of 40 was determined based on the Youden index, which effectively stratifies individuals into groups of good and intermediate DM control versus poor DM control. The Malaysian CPG emphasises individualised targets for the HbA1c level rather than a universal standard. For instance, a target of <6.5% is recommended for newly diagnosed or younger patients, whereas a target of 7.1%–8.0% is suggested for older patients with a shorter life expectancy. In this study, however, a uniform HbA1c reference level was applied across the entire population instead of tailoring targets to subgroups. The HbA1c reference level was adopted from the study by Mori et al.^7^ Conversely, the serum creatinine level was used as a numerical value instead of converting it to the eGFR because the creatinine level directly reflects the renal function status and was readily available in the dataset without further transformations. By using the serum creatinine level as a direct and continuous measure, the study minimised data transformation errors and ensured consistency with the available dataset.

Ethnic classifications in Malaysia are diverse, with several ethnic groups represented by substantially small populations. To facilitate meaningful analysis using multinomial logistic regression, we consolidated ethnicity into two categories: Malay and non-Malay. This approach addressed the challenges posed by small populations in certain ethnic groups, ensuring robust and interpretable analysis.

### DM control classification

DM control was classified based on the HbA1c level. The HbA1c level reflects the average plasma glucose level over the preceding 3 months.^12^ The United Kingdom Prospective Diabetes Study and the Diabetes Control and Complications Trial clearly demonstrated the correlation of an increasing HbA1c level with an increased risk of complications^12^ In this study, an HbA1c level of <7% indicated good DM control; HbA1c level of 7%-8%, intermediate DM control; and HbA1c level of ≥8%, poor DM control.^7^

### Statistical analysis

All analyses were performed using SPSS version 29 (IBM Corp., Armonk, NY, USA). A twosided analysis was utilised to determine statistical significance, with a P-value of less than 0.05 as the criterion.

In the descriptive analysis, categorical data were expressed as frequencies (percentages) and continuous data as means ± standard deviations. In the univariate analysis, unadjusted odds ratios (ORs) for the association between BMI and different DM control classifications were analysed using ANOVA for trend for continuous data. Categorical data were analysed using the linear-by-linear association chi-square test. Interactions, outliers and multicollinearities were also examined. The chi-square goodness-of-fit test was used to examine model fitness.

In the multivariable analysis, adjusted ORs for the association between BMI and different DM control classifications were analysed using multinomial logistic regression. Model 0 was unadjusted for BMI. Later models were adjusted based on previous models. Model 1 was adjusted for the sociodemographic characteristics; Model 2, medical illnesses; Model 3, lipid-lowering medications; Model 4, ticlopidine and acetylsalicylic acid; Model 5, antihypertensive medications; Model 6, glucose-lowering medications; and Model 7, biochemical readings to measure the trend of risk measurement across different DM control classifications.

## Results

A total of 1955 patients were included in this study. Their characteristics are shown in Table 1. The majority of the patients were 40 years old and above (97.0%), Malay (76.0%) and women (62.1%). The mean duration of DM was more than 8 years. The mean systolic and diastolic blood pressures were higher than the recommended value (below 130/75 mmHg) for patients with DM. The HbA1c level was also higher than the level highlighted in the 6th edition Malaysian CPG for Diabetes Mellitus (6.5%).^11^ The fasting serum lipid profiles, which included the TC, LDL and TG levels, were higher than the levels recommended by the Malaysian CPG for the Management of Dyslipidaemia in Type 2 Diabetes Mellitus. Conversely, the mean HDL level was more than 1.2 mmol/L.

**Table 1:** Characteristics of the patients with type 2 Diabetes Mellitus (T2DM) from Muar District Health Office, Johor (N=1955).

Variable	Frequency, n (%)	Mean ± SD
**a) Sociodemographic characteristics:**		
Age		
<40 years	59 (3.0)	
≥40 years	1896 (97.0)	
Sex		
Male	740 (37.9)	
Female	1215 (62.1)	
Ethnicity		
Malay	1485 (76.0)	
Non-Malay	470 (24.0)	
Smoking status		
Yes	110 (5.6)	
No	1845 (94.4)	
**b) Anthropometric measurements and medical illnesses:**		
BMI, kg/m^2^		28.11+5.70
DM duration, year		8.55+5.83
Hypertension		
Systolic blood pressure (mmHg)		142.52+17.20
Diastolic blood pressure (mmHg)		77.40+10.96
Hypertension status		
Yes	1621 (82.9)	
No	334 (17.1)	
Dyslipidaemia status		
Yes	1637 (83.7)	
No	318 (16.3)	
**c) Biochemical profile:**		
HbA1c level (%)		7.96+1.95
Total cholesterol level (mmol/L)		4.89+1.16
Triglyceride level (mmol/L)		1.77+1.13
HDL level (mmol/L)		1.36+0.37
LDL level (mmol/L)		2.74+1.02
Serum creatinine level (mmol/L)		86.12+57.84
Proteinuria		
Yes	1346 (68.8)	
No	609 (31.2)	
**d) Medication:**		
Metformin		
Yes	1719 (87.9)	
No	236 (12.1)	
Sulfonylurea		
Yes	824 (42.1)	
No	1131 (57.9)	
Insulin		
Yes	542 (27.7)	
No	1413 (72.3)	
Acetylsalicylate acid		
Yes	272 (13.9)	
No	1683 (86.1)	
Ticlopidine		
Yes	22 (1.1)	
No	1933 (98.9)	
ACE inhibitor		
Yes	1065 (54.5)	
No	890 (45.5)	
Angiotensin-receptor blocker		
Yes	194 (9.9)	
No	1761 (90.1)	
Beta-blocker		
Yes	540 (27.6)	
No	1415 (72.4)	
Calcium-channel blocker		
Yes	1163 (59.5)	
No	792 (40.5)	
Diuretic		
Yes	288 (14.7)	
No	1667 (85.3)	
Alpha-blocker		
Yes	65 (3.3)	
No	1890 (96.7)	
Statin		
Yes	1739 (89.0)	
No	216 (11.0)	
Fibrate		
Yes	8 (0.4)	
No	1947 (99.6)	
Alpha-glucosidase inhibitor		
Yes	12 (0.6)	
No	1943 (99.4)	

**Figure 1:**
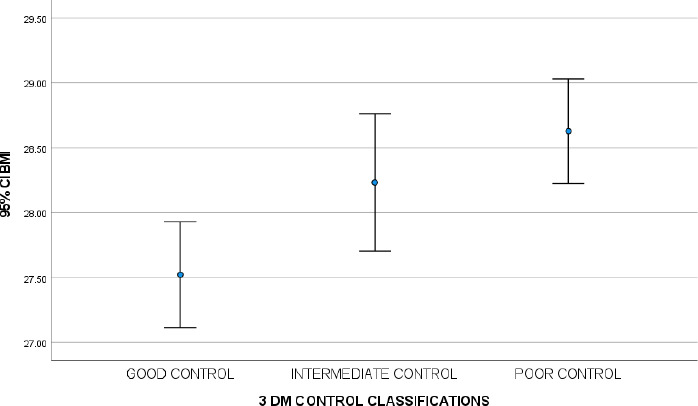
Trend of the body mass index (BMI) across the Diabetes Mellitus (DM) control classifications.

Among the patients, 759 (38.8%, 95% CI=36.7, 41.0) had good DM control; 433 (22.2%, 95% CI=20.3, 24.0), intermediate DM control; and 763 (39.0%, 95% CI=36.7, 41.2), poor DM control. Figure 1 illustrates the trend of the mean BMI across the DM control classifications. The mean BMI increased from good to poor DM control classifications. The mean BMI was 27.52±5.71 kg/m^2^ among the patients with good DM control, 28.23±5.62 kg/m^2^ among the patients with intermediate DM control and 28.63±5.68 kg/m^2^ among the patients with poor DM control (P-value for trend: <0.001).

Tables 2 and 3 show the univariate analysis of the categorical and numericalvariables stratified by the DM control classifications (good, intermediate and poor), respectively.

**Table 2:** Univariate trend analysis of the categorical variables stratified by the DM control classifications (N=1955).

Variable	DM control classification			Total n (%)	χ^2^ (df)	P-value for trend
	Good (n=759) n (%)	Intermediate (n=433) n (%)	Poor (n=763) n (%)			
**a) Sociodemographic characteristics**						
Age:						
<40 years ≥40 years	13 (22.0) 746 (39.3.3)	10 (16.9) 423 (22.3)	36 (61.0) 727 (38.3)	59 (100.0) 1896 (100.0)	11.745 (1)	<0.001^*^
Sex:						
Male Female	282 (38.1) 477 (39.3)	174 (23.5) 259 (21.3)	284 (38.4) 479 (39.4)	740 (100.0) 1215 (100.0)	0.001 (1)	0.980
Ethnicity:						
Malay Non-Malay	535 (36.0) 224 (47.7)	328 (22.1) 105 (22.3)	622 (41.9) 141 (30.0)	1485 (100.0) 470 (100.0)	25.351 (1)	<0.001^*^
Smoking status:						
Yes No	39 (35.5) 720 (39.0)	26 (23.6) 407 (22.1)	45 (40.9) 718 (38.9)	110 (100.0) 1845 (100.0)	0.412 (1)	0.521
**b) Medical illnesses**						
Hypertension status:						
Yes No	666 (41.1) 93 (27.8)	358 (22.1) 75 (22.5)	597 (36.8) 166 (49.7)	1621 (100.0) 334 (100.0)	24.244 (1)	<0.001^*^
Dyslipidaemia status:						
Yes No	638 (39.0) 121 (38.1)	355 (21.7) 78 (24.5)	644 (39.3) 119 (37.4)	1637 (100.0) 318 (100.0)	0.034 (1)	0.854
**c) Biochemical profile**						
Proteinuria:						
Yes No	472 (35.1) 287 (47.1)	293 (21.8) 140 (23.0)	581 (43.2) 182 (29.9)	1346 (100.0) 609 (100.0)	34.564 (1)	<0.001^*^
**d) Medications**						
Metformin:						
Yes No	655 (38.1) 104 (44.1)	383 (22.3) 50 (21.2)	681 (39.6) 82 (34.7)	1719 (100.0) 236 (100.0)	3.127 (1)	0.077
Sulfonylurea:						
Yes No	254 (30.8) 505 (44.7)	208 (25.2) 225 (19.9)	362 (43.9) 401 (35.5)	824 (100.0) 1131 (100.0)	30.440 (1)	<0.001^*^
Insulin:						
Yes No	71 (13.1) 688 (48.7)	102 (18.8) 331 (23.4)	369 (68.1) 394 (27.9)	542 (100.0) 1413 (100.0)	288.876 (1)	<0.001^*^
Alpha-glucosidase inhibitor:						
Yes No	2 (16.7) 757 (39.0)	3 (25.0) 430 (22.1)	7 (58.3) 756 (38.9)	12 (100.0) 1943 (100.0)	2.665 (1)	0.103
Acetylsalicylate acid:						
Yes No	114 (41.9) 645 (38.3)	67 (24.6) 366 (21.7)	91 (33.5) 672 (39.9)	272 (100.0) 1683 (100.0)	3.042 (1)	0.081
Ticlopidine:						
Yes No	12 (54.5) 747 (38.6)	4 (18.2) 429 (22.2)	6 (27.3) 757 (39.2)	22 (100.0) 1933 (100.0)	2.157 (1)	0.142
ACE inhibitor:						
Yes No	388 (36.4) 371 (41.7)	233 (21.9) 200 (22.5)	444 (41.7) 319 (35.8)	1065 (100.0) 890 (100.0)	7.670 (1)	0.006^*^
Angiotensin-receptor blocker:						
Yes No	83 (42.8) 676 (38.4)	56 (28.9) 377 (21.4)	55 (28.4) 708 (40.2)	194 (100.0) 1761 (100.0)	5.924 (1)	0.015^*^
Beta-blocker:						
Yes No	232 (43.0) 527 (37.2)	112 (20.7) 321 (22.7)	196 (36.3) 567 (40.1)	540 (100.0) 1415 (100.0)	4.522 (1)	0.033^*^
Calcium-channel blocker:						
Yes No	486 (41.8) 273 (34.5)	249 (21.4) 182 (23.2)	428 (36.8) 335 (42.3)	1163 (100.0) 792 (100.0)	9.934 (1)	0.002^*^
Diuretic:						
Yes	108 (37.5)	59 (20.5)	121 (42.0)	288 (100.0)	0.805 (1)	0.370
No	651 (39.1)	374 (22.4)	642 (38.5)	1667 (100.0)		
Alpha-blocker:						
Yes	32 (49.2)	12 (18.5)	21 (32.3)	65 (100.0)	2.532 (1)	0.112
No	727 (38.5)	421 (22.3)	742 (39.3)	1890 (100.0)		
Statin:						
Yes	666 (38.3)	389 (22.4)	684 (39.3)	1739 (100.0)	1.394 (1)	0.238
No	93 (43.1)	44 (20.4)	79 (36.6)	216 (100.0)		
Fibrate:						
Yes	3 (37.5)	3 (37.5)	2 (25.0)	8 (100.0)	0.166 (1)	0.683
No	756 (38.8)	430 (22.1)	761 (39.1)	1947 (100.0)		

* Statistically significant at α=0.05

Statistical analysis: Linear-by-linear association

**Table 3:** Univariate trend analysis of the numerical variables stratified by the DM control classifications (N=1955).

Variable	DM control classification (control)			Total n (%)	F (df)	P-value for trend
	Good (n=759) n (%)	Intermediate (n=433) n (%)	Poor (n=763) n (%)			
**a) Sociodemographic characteristics**						
BMI, kg/m^2^	27.52+5.71	28.23+5.62	28.63+5.68	28.11+5.70	7.34 (1, 1952)	<0.001^*^
**b) Medical illnesses**						
DM duration, year	7.49+5.65	8.54+5.90	9.62+5.77	8.55+5.83	26.06 (1, 1952)	<0.001^*^
Systolic BP, mmHg	141.79+16.60	142.36+16.79	143.33+17.99	142.52+17.20	1.545 (1, 1952)	0.214
Diastolic BP, mmHg	75.88+10.36	77.31+11.09	78.97+11.25	77.40+10.96	15.396 (1, 1952)	<0.001^*^
**c) Biochemical profile**						
HbA1c level, %	6.27+0.44	7.41+0.29	9.96+1.58	7.96+1.95	2495.778 (1, 1952)	<0.001^*^
Total cholesterol level	4.71+1.05	4.82+1.09	5.11+1.25	4.89+1.16	24.396 (1, 1952)	<0.001^*^
TG level	1.58+0.88	1.77+1.13	1.95+1.32	1.77+1.13	21.074 (1, 1952)	<0.001^*^
HDL level	1.42+0.39	1.33+0.34	1.32+0.35	1.36+0.37	14.509 (1, 1952)	<0.001^*^
LDL level	2.59+0.95	2.71 + 1.00	2.92+1.07	2.74+1.02	20.437 (1, 1952)	<0.001^*^
Serum creatinine level	89.53+64.83	87.56+55.34	81.74+51.28	86.12+57.84	3.709 (1, 1952)	0.025^*^

* Statistically significant at α=0.05

Statistical analysis: One-way ANOVA for trends

For the categorical variables, there were significant trends in the classification of DM control for age (P<0.001), ethnicity (P<0.001), hypertension status (P<0.001), proteinuria (P<0.001) and medications such as sulfonylurea (P<0.001) and insulin (P<0.001). Additionally, the trends for ACE inhibitors (P=0.006), angiotensin-receptor blockers (P=0.015), beta-blockers (P=0.033) and calcium-channel blockers (P=0.002) were significant. The other variables showed no significant trends.

For the numerical variables, there were significant trends in the classification of DM control for BMI (P<0.001), DM duration (P<0.001), diastolic blood pressure (P<0.001), TC level (P<0.001), TG level (P<0.001), HDL level (P<0.001), LDL level (P<0.001) and serum creatinine level (P=0.025).

**Table 4:** ORs and adjusted ORs of BMI according to the DM control classifications (N=1955).

Model	DM control classification			P-value	R^2^ (df)
	Good (n=759) n (%)	Intermediate (n=433) n (%)	Poor (n=763) n (%)		
Model 0: Unadjusted					
BMI	1	1.023 (1.002, 1.045)^*^	1.035 (1.017, 1.054)^*^	<0.001	0.008
Model 1: Adjusted for the sociodemographic characteristics (age, sex and ethnicity)					
BMI	1	1.020 (0.998, 1.042)	1.025 (1.006, 1.044)^*^	0.023	0.023
Model 2: Adjusted for underlying medical illnesses (DM duration, hypertension status and dyslipidaemia status) and smoking status based on Model l					
BMI	1	1.026 (1.003, 1.049)^*^	1.035 (1.014, 1.056)^*^	0.003	0.101
Model 3: Adjusted for lipid-lowering drugs (statin and fibrate) based on Model 2					
BMI	1	1.025 (1.002, 1.048)^*^	1.034 (1.013, 1.055)^*^	0.004^*^	0.104
Model 4: Adjusted for antiplatelet drugs (acetylsalicylic acid and ticlopidine) based on Model 3					
BMI	1	1.025 (1.002, 1.048)	1.033 (1.013, 1.054)^*^	0.005^*^	0.107
Model 5: Adjusted for glucose-lowering drugs (metformin, sulfonylurea, alpha-glucosidase inhibitor and insulin) based on Model 4					
BMI	1	1.021 (0.998, 1.045)	1.028 (1.006, 1.051)^*^	0.034^*^	0.241
Model 6: Adjusted for antihypertensive drugs (ACE inhibitor, angiotensin-receptor blocker, beta-blocker, calcium-channel blocker, diuretic and alpha-blocker) based on Model 5					
BMI	1	1.022 (0.999, 1.046)	1.031 (1.008, 1.054)^*^	0.022^*^	0.249
Model 7: Adjusted for the biochemical profile (TG level, total cholesterol level, LDL level, proteinuria and serum creatinine level) based on Model 6					
BMI	1	1.018 (0.994, 1.043)	1.031 (1.008, 1.055)^*^	0.029^*^	0.288
Model 8: Adjusted for the biochemical profile (HDL level) based on Model 7					
BMI	1	1.016 (0.992, 1.040)	1.028 (1.004, 1.052)^*^	0.066	0.293
Model 9: Adjusted for the systolic and diastolic blood pressures based on Model 8					
BMI	1	1.009 (0.985, 1.034)	1.013 (0.990, 1.038)	0.525	0.305

ORs and adjusted ORs (95% CIs) compared to patients with good DM control

* Statistically significant at α=0.05

Pseudo R^2^=0.305

Statistical test: Hierarchical multinomial logistic regression

Based on table 4, in Model 0, which was unadjusted, there was a significant trend in BMI across the DM control classifications (P<0.001). In Models 1 and 2, after adjusting for the sociodemographic characteristics (age, sex and ethnicity) and underlying medical illnesses along with smoking status, we found a significant trend in BMI across the DM control classifications (P=0.003-0.023). The trend odds increased from good to poor DM control. In Models 3-6, there was a significant trend in BMI across the DM control classifications even after adjusting for medications (P=0.003-0.034). The trend odds increased from good to poor DM control. In Model 7, after adjusting for the biochemical profile (TG level, TC level, LDL level, proteinuria and serum creatinine level), we observed a significant trend in BMI across the DM control classifications (P=0.029). There was a significant difference between poor and good DM control. The trend odds still increased from good to poor DM control. In Models 8 and 9, after adjusting for the HDL level and systolic and diastolic blood pressures, we noted a non-significant trend in BMI across the DM control classifications (P=0.066-0.525).

In the poor DM control group, the adjusted OR increased from 1.025 to 1.035 after adjusting for underlying medical illnesses and smoking status. The adjusted OR further decreased with the presence of medications in Models 3-5 including lipid-lowering drugs, antiplatelet drugs and glucose-lowering drugs. However, the OR increased after adjustment for antihypertensive drugs in Model 6 and remained static after adjustment for the biochemical profile except for the HDL level in Model 7. In Models 8 and 9, the adjusted OR for poor DM control decreased, but this change was not significant.

## Discussion

Malaysia has adopted a yearly audited programme by the NDR with a target of more than 30% of randomly selected patients having an HbA1c level of less than 6.5%.^10^ In this study, the distribution of patients with DM is almost the same as that in other studies.^13-15^ Most patients were women and older adults.^13-15^ However, Chen et al.^16^ found that patients had a normal BMI. The duration of DM is longer in this study than in other studies.^17,18^ Conversely, the HbA1c level is slightly lower than that in the study by Bramlage et al.^17^

The systolic and diastolic blood pressures in this study are slightly higher than those in other studies.^17,18^ The systolic blood pressure was higher than the targeted value, but the diastolic blood pressure remained in good control.^19^ The biochemical profile, such as the fasting serum lipid level, was higher than the recommended range for patients with T2DM except for the HDL level. However, the biochemical profile in this study is almost the same as that in another study.^15^

This study found that BMI was significantly associated with the DM control classifications. The mean BMI increased from good to poor DM control. The trend showed an increase in BMI (P<0.001). The association was independent of the sociodemographic characteristics (age, sex and ethnicity), blood pressure (systolic and diastolic), underlying medical conditions (hypertension and dyslipidaemia), smoking status, medications (glucose-lowering drugs, lipid-lowering drugs, antihypertensive drugs and antiplatelet drugs) and biochemical profile.

There was a strong relationship between BMI and diabetes and insulin resistance.^20^ Hyperinsulinaemia and insulin resistance are closely linked with central obesity. Visceral fat raises the risk of diabetes by promoting insulin resistance. This increased risk from intraabdominal fat for diabetes and other metabolic conditions could be due to the higher number of fat cells in abdominal tissue, greater blood flow, more receptors for cortisol and testosterone and increased catecholamine-induced lipolysis compared to subcutaneous fat. Additionally, there is a significant rise in the flow of non-esterified fatty acids to the liver in individuals with abdominal obesity. There is ample evidence that abdominal obesity causes insulin resistance and is a critical component of metabolic syndrome, leading to poor glycaemic control.^21^

There was a significant trend between sex and the DM control classifications. A study conducted in Oman reported that sex was associated with poor DM control,^13^ while another study performed in Malaysia found that the female sex was a predictor for poor DM control.^15^ A review report in 2019 found that the incidence of poor glycaemic control among women was higher because they experience more difficulties in achieving adequate glycaemic control compared with men.^22^ Possible reasons for the different outcomes between men and women were differences in glucose and energy homoeostasis (hormones and visceral adipose tissues),^23^ treatment response and psychological factors (e.g. disease acceptance).^24^ Despite the potential effect of sex on glycaemic control, there are currently no specific treatment guidelines for men and women with T2DM.

The OR of BMI increased after being adjusted for the duration of DM across the DM control classifications compared to good DM control. The duration since DM diagnosis was also another predictor of poor glycaemic control. The patients who had DM for a longer duration (more than 7 years) were more likely to have poor glycaemic control. The study conducted by Mamo et al.^25^ in Ethiopia also indicated that patients who had diabetes for 7 years were more likely to have poor glycaemic control. In line with these results, Haghighatpanah et al.^26^ revealed that patients who were diagnosed with diabetes more than 10 years ago were more likely to have poor glycaemic control than those with a duration of less than or equal to 10 years. This could be due to the progressive impairment of insulin secretion with time by fi-cells, increase in insulin resistance and sudden decrease in insulin secretion.^27^

The OR of BMI decreased after being adjusted for lipid-lowering drugs across both intermediate and poor DM control compared to good DM control (P=0.003). A previous study found that statins, which are lipid-lowering drugs, led to poor DM control.^28^ However, in this study, most of the patients concomitantly used lipid-lowering drugs with other medications. Research has shown that concomitant use of lipid-lowering drugs such as statins with metformin significantly improves glycaemic control.^29^ After adjusting further for antiplatelet drugs, we observed that the OR of BMI decreased significantly across both intermediate and poor DM control compared to good DM control (P=0.005). A study conducted in the USA found that acetylsalicylic acid inhibited the activity of serine kinase IKKp, which could help reduce insulin resistance and improve glucose tolerance in patients with T2DM.^30^

The OR of BMI significantly decreased further after adjusting for glucose-lowering drugs in the poor DM control group (P=0.034) but did not significantly decrease in the intermediate DM control group, both compared to the good DM control group. This suggests that treating patients with these medications can reduce the likelihood of progression of DM control classification. Several studies have indicated that glucose-lowering drugs impact BMI and improve glycaemic control.^31,32^ After adjusting further for antihypertensive medications, we found that the OR of BMI significantly increased in the poor DM control group compared to the good DM control group (P=0.022) but did not significantly increase in the intermediate DM control group. These findings can be explained by the distribution of medications used in this study. Diuretics and beta-blockers were found to be associated with increased weight gain and were diabetogenic.^33^ However, other medications such as calcium-channel blockers did not significantly reduce the HbA1c level and were positively associated with fi-cell function in the patients with hypertension and T2DM.^34^

The OR of BMI decreased in the intermediate DM control group (P=0.029), but this change was not significant. The OR did not change in the poor DM control group compared to the good DM control group after adjusting for the biochemical profile. This is because higher TG, TC and LDL levels lead to abnormality towards the HbA1c level.^15,35^ Deficiencies in lipoprotein lipase, an insulin-sensitive enzyme, might explain the abnormal levels of remnant particles in insulin resistance.^36^ However, when the variable was adjusted for the HDL level, the OR decreased significantly in the intermediate DM control group. According to Femlak et al.,^37^ HDL has properties that can be compromised by the oxidative modification and glycation of the HDL protein as well as the transformation of the HDL proteome into proinflammatory protein.

There was no significant association found between DM control and BMI after adjusting for blood pressure (P=0.525). A previous RCT^38^ found that an intensive blood pressure treatment was not associated with an increased risk of incident DM but significantly increased the risk of impaired fasting blood sugar. Therefore, the risks and benefits of intensive blood pressure targets should be factored into individualised patient treatment goals.

### Limitations

There are some limitations of this study. The study was conducted using secondary data, which may not provide all relevant information such as dietary intake and physical activities. Based on the theoretical framework, several variables were not present in the NDR because the system has been developed to answer different objectives.^39^ The use of secondary data also lacked control over the quality of the data because the researcher did not participate in the data collection processes. Additionally, the cross-sectional observational nature of the study has limitations in terms of the analyses of causal inference, as this study design is unable to measure temporality based on Bradford Hill’s criteria.^40^

The creatinine level was identified as a significant variable for DM classification. However, the analysis could not determine its specific weightage on the outcome. Additionally, since multinomial logistic regression was used with all parameters included in a single model, stratification of the HDL level based on sex was not possible. Furthermore, the absence of data on waist circumference in the database prevented the assessment of its correlation with DM control status.

## Conclusion

There is an association between BMI, the creatinine level and DM control classification even after adjusting for several confounding factors. Medications such as glucose-lowering drugs, ticlopidine, acetylsalicylic acid and statins should be initiated earlier to prevent worsening of DM control. However, as the study design does not allow for the assessment of causality or progression over time, the findings should be interpreted as descriptive associations rather than as evidence of cause-and-effect relationships. Future longitudinal studies are recommended to explore the temporal impact of these factors on DM progression and control. From the perspective of healthcare providers (e.g. pharmacists), it is recommended that they play a role in explaining the benefits and risks of each medication to prevent decreased compliance among patients with T2DM. Further, effective weight management programmes should be emphasised to patients with diabetes to reduce the impact of diabesity. Additionally, stakeholders must ensure that patients have adequate access to necessary medications.

## Data sharing statement

The datasets generated and/or analysed during the current study are not publicly available but are available from the corresponding author upon reasonable request.

**How does this paper make a difference in general practice?**

- There are associations and significant trends observed between BMI, the creatinine level and medications such as glucose-lowering drugs, ticlopidine, acetylsalicylic acid and statins relative to DM control classifications after adjusting for several confounders.
- Focus on medication compliance, healthcare providers’ role during medication consultation and stakeholders’ role in maintaining drug supplementation is needed.
